# Global insight into understanding wheat yield and production through Agro-Ecological Zoning

**DOI:** 10.1038/s41598-023-43191-x

**Published:** 2023-09-23

**Authors:** Amir Dadrasi, Mehrdad Chaichi, Alireza Nehbandani, Elias Soltani, Ahmad Nemati, Fatemeh Salmani, Moslem Heydari, Ali Reza Yousefi

**Affiliations:** 1 Department of Agronomy, Agriculture College, Vali-e-Asr University of Rafsanjan, Rafsanjan, Iran; 2 Department of Seed and Plant Improvement Research, Hamadan Agriculture and Natural Resources, Research and Education Center, Agriculture Research, Education and Extension Organization, Hamadan, Iran; 3 Department of Plant Production, Gorgan University of Agricultural Sciences, Gorgan, Iran; 4 Department of Agronomy and Plant Breeding Sciences, College of Aburaihan, University of Tehran, Tehran, Iran; 5 Department of Plant Production and Genetics, University of Zanjan, Zanjan, Iran

**Keywords:** Ecology, Plant sciences, Climate sciences

## Abstract

Global food security requires food production to be increased in the future decades. Agro-Ecological Zones (AEZ) methodology is a successful approach used in land evaluation studies to support sustainable agricultural development. This approach can facilitate finding suitable areas for wheat production on a global scale. This study was conducted based on a climate zone map, soil data, wheat cultivation area, yield, and production under irrigated and rainfed conditions, worldwide. The results for irrigated wheat indicated that there is an area of 59.5 Mha with an average yield of 4.02 t/ha which leads to the production of about 239.6 Mt of grain yield. Furthermore, climate zones (CZs) of 8002, 5203, 5302, 5403, and 8102 have the highest wheat production with an average of 20.7, 14.2, 13.3, 11.8, 11.5 Mt, respectively. The highest amount of irrigated wheat production has been achieved in soil type code 17 (Loam LF120), which has a cultivation area of around 23.6 Mha and a production of about 106.8 Mt. Rainfed wheat production is 410 Mt, and the cultivation area is 160.2 Mha. The highest rainfed wheat production with an average of 17 Mt was related to the 3702 CZ, followed by the other CZs (3802, 1303, 1203, 3602, 4602, etc.). The soil codes 11 (Loam HF120), 10 (Loam HF180), and 14 (Loam MF120) showed the highest rainfed wheat production. The findings of this study can be useful for agricultural scientists, authorities, and decision-makers around the world to find suitable lands to expand wheat cultivation and also to find new locations for increasing global wheat production to feed the increasing population in the world.

## Introduction

The Agro-Ecological Zoning (AEZ) approach is an effective tool utilized in land evaluation to ensure the sustainability of agricultural development. It assesses resource limitations and opportunities based on crop eco-physiological properties, climate and soil requirements to determine the suitability and production potential for specific crops with appropriate inputs and management practices^1^. To achieve optimal and sustainable agricultural production, the environmental and climatic conditions must be defined in terms of their capabilities and limitations. Similar regions are then grouped together based on these conditions, resulting in a climatic classification or zoning^2^. This zoning, which takes into account both climatic and soil properties, is known as Agro-Ecological Zoning and can be used to evaluate the potential and limitations of land resources for improving agricultural production^3–5^.

Advantages of Agro-Ecological Zoning include: (1) Identification of similar or different regions for specific purposes, (2) Enhancement of technology transfer and research results, (3) Determination of crop stress periods and, (4) Focusing of research efforts^3,6,7^. The concept of Agro-Ecological Zoning was first proposed by the Food and Agriculture Organization (FAO) in the 1970s^8^, as a means of evaluating lands and determining the agricultural production potential at a regional and national level^9^.

The determination of climatic zones is a crucial aspect in ensuring the optimal and sustainable production of crops. Several methods for agro-climatic zoning exist, including FAO, CGIAR-TAC, Prentice, Pappadakis Köppen-Geiger, Holdridge, GAEZ-LGP, HCAEZ, SAGE, GLI, and Gens^10,11^. A recent addition to these methods is the Global Yield Gap Atlas extrapolation (GYGA) method, which is a simplified combination of existing zoning methods. Unlike the SAGE and GLI methods, which utilize a base temperature specific to each crop, the GYGA-ED method uses a single base temperature (0 °C), resulting in a uniform climatic zone set for all crops and making it easier to use. Both GYGA-ED and GEnS approaches are not specific to a particular plant and display high uniformity within each climatic group^10^. The GYGA-ED method has been shown to be the best data sources for estimation of potential yield and yield gap of crops such as potatoes and soybean, as demonstrated by the studies of Dadrasi et al.^12^ and Nehbandani et al.^13^.

The GYGA approach integrates a suite of essential parameters, each playing a vital role in determining crop growth and performance. Among these key parameters, growing degree days (GDD), temperature seasonality, and aridity index stand out as crucial factors shaping agro-ecological zones and agricultural production. Growing degree days, a measure of accumulated heat units, quantifies the thermal conditions experienced by a crop during its growth cycle. These thermal conditions significantly influence the crop's development and ultimately affect its yield potential. By incorporating GDD in AEZ studies, researchers can assess the suitability of different regions for specific crops, as well as predict optimal planting and harvesting times. Moreover, understanding GDD variations across regions aids in identifying areas where extended growing seasons may offer opportunities for multiple cropping or crop diversification strategies^14^.

Temperature seasonality, another vital parameter in the GYGA procedure, characterizes the variation in temperature over the course of a year. High temperature seasonality implies significant fluctuations in temperatures between seasons, potentially impacting crop growth and development. Areas experiencing extreme temperature variations might be less conducive to cultivating certain crops, as the abrupt changes can lead to stress and reduced yields. By considering temperature seasonality in AEZ studies, researchers can better discern the climatic suitability of specific regions for various crops, thereby fostering more efficient land use and resource allocation^1^.

The aridity index, which assesses the balance between precipitation and evapotranspiration, serves as a pivotal determinant of water availability and drought risk in agro-ecological zones. Understanding the aridity index is crucial for optimizing irrigation practices, selecting drought-resistant crop varieties, and mitigating water scarcity challenges. AEZ studies that account for the aridity index provide valuable insights into the feasibility of agricultural activities in arid and semi-arid regions, guiding policymakers and farmers in making informed decisions related to water management and sustainable crop production^1^.

Wheat (*Triticum aestivum*) is considered a vital food crop globally^15^ and its significance is increasing due to political issues between Russia and Ukraine. The cultivation of wheat takes place in both an irrigated and rainfed system, according to Deihimfard et al.^16^. In regions with abundant rainfall, the crop is grown as rainfed, while in arid regions, it is grown using irrigation. Additionally, wheat is considered a valuable cash crop due to its high yield per unit area, its ability to thrive in a temperate climate with a moderate growing season, and the production of high-quality, versatile flour^17^. In 2020, the global production of wheat was 760 million tons, with China, India, and Russia accounting for 41% of total production^18^. To ensure appropriate and sustainable production of wheat, it is important to understand the environmental and climatic conditions of its cultivation, including the planting date, cultivar ripening time, plant density, soil and meteorological data. However, acquiring this information can be time-consuming and costly. Agro-climatic zoning can provide access to this information on a large scale, as demonstrated by Fischer et al.^1^.

This study aims to determine the amount of production and cropland area for both irrigated and rainfed wheat in each soil and climate zone, as well as their combination, in order to identify the main regions of wheat cultivation worldwide. Additionally, the study can determine the extent to which rainfed and irrigated wheat cultivation can be replaced, if necessary, due to factors such as climate change.

## Materials and methods

In this research, an analysis was conducted on the existing climate and soil zones in irrigated and rainfed wheat lands around the world. The frequency of production, cultivation area, and yield were determined for each zone. A map of the climatic and soil zones was also created. By combining the climate and soil zones, agro-ecological zones for irrigated and rainfed wheat lands were established. The results from the agro-ecological zones were analyzed, and the frequency percentage for each zone was calculated. A map of the agro-ecological zones was also generated.

### GYGA climate map

The GYGA Climate Map allowed us to divide the global wheat cultivation areas into distinct climatic zones based on the three climatic factors mentioned earlier. The calculation of Growing Degree Days (GDD) involved considering the average daily temperature throughout the year and categorizing the zones into ten classes based on GDD values. The Temperature Seasonality was determined by calculating the standard deviation of average monthly temperatures and classifying the zones into three categories. Lastly, the Annual Aridity Index (AI) was obtained by calculating the ratio of average annual rainfall to average annual evaporation and dividing the zones into ten classes.

On this map, the climatic zones are divided based on the data from the three factors listed on http://www.yieldgap.org/web/guest/cz-ted, which are:

1. The growing degree days (GDD) calculated with a base temperature of 0 °C (Eq. 1).
2. Temperature seasonality
3. The Annual aridity index (AI)

1 $$GDD=\sum_{i}^{n}{t}_{i}$$

where, GDD is the unit of temperature throughout the year in terms of day degrees; n, the number of days during the year, which is 365 days in normal years and 366 days in leap years; i, day of the year; t_i_, the average daily temperature on a day i. If the average daytime temperature of the year is below zero degrees Celsius, t_i_ is considered zero for that day. Obviously, for a climatic zone, the higher GDD value indicates that the average temperature of that climatic zone is higher throughout the year.

The temperature seasonality is the standard deviation of average monthly temperatures that is obtained from Eq. (2):

2 $$\text{Temperature Seasonality}=\sqrt{\sum_{m}^{12}\frac{{({t}_{m}-{t}_{avr})}^{2}}{12}}$$

where, temperature seasonality is the coefficient of temperature fluctuations throughout the year; t_m_: the average temperature in the month m in degrees Celsius; t_avr_: the average temperature throughout the year in degrees Celsius. The larger seasonal temperature fluctuations coefficient for a climatic zone shows that temperature fluctuations are higher throughout the year in that climatic zone. In simpler terms, the difference between the coldest and warmest months of the year is greater.

The third index used for zoning by the extrapolation method of the Global Atlas of yield gap is an aridity index derived from Eq. (3):

3 $$AI=\frac{MAP}{MAE}$$

where AI is annual aridity index; MAP: average annual rainfall in millimeters; MAE: average annual evaporation in millimeters. according to this equation, in a climatic zone, the smaller the amount of AI, the drier the zone.

The process of zoning began by dividing the entire globe into pixels. Each pixel measured around 5 min, which roughly translated to 10 × 10 km. In order to calculate the subsequent results, only pixels that had a minimum coverage of 0.5% with major crops such as corn, rice, wheat, sorghum, millet, barley, soybean, cassava, potatoes, sweet potatoes, bananas, peanuts, beans and other legumes, sugar beets, and sugarcane were considered (http://www.yieldgap.org/web/guest/cz-ted).

The calculation of the values of the mentioned variables is performed for every pixel. Afterwards, categorization is performed based on the classifications assigned to each of these variables (as shown in Table 1) and the combination of these three variables. Ten categories have been established for the Growing Degree Days, ten categories for the Annual Aridity Index, and three categories for the Temperature Seasonality.

**Table 1:** Classification of climate variables in the global atlas of yield gap using the extrapolation method.

GYGA code	GDD*	GYGA code	Aridity index**	GYGA code	Temperature seasonality***
1000	0–2670	0	0–0.2695	1	0–3.832
2000	2671–3169	100	0.2696–0.3893	2	3.833–8.355
3000	3170–3791	200	0.3894–0.4791	3	> 8.356
4000	3792–4829	300	0.4792–0.5689		
5000	4830–5949	400	0.5690–0.6588		
6000	5950–7111	500	0.6589–0.7785		
7000	7112–8564	600	0.7786–0.8685		
8000	8565–9311	700	0.8686–0.10181		
9000	9312–9850	800	0.10182–12,876		
10,000	> 9851	900	> 12,877		

*Calculation of the GDD based on the base temperature of zero degrees Celsius.

**Ratio of annual rainfall to annual evaporation potential.

***The amount of standard deviation of the monthly temperature from the average annual temperature.

**Figure 1:**
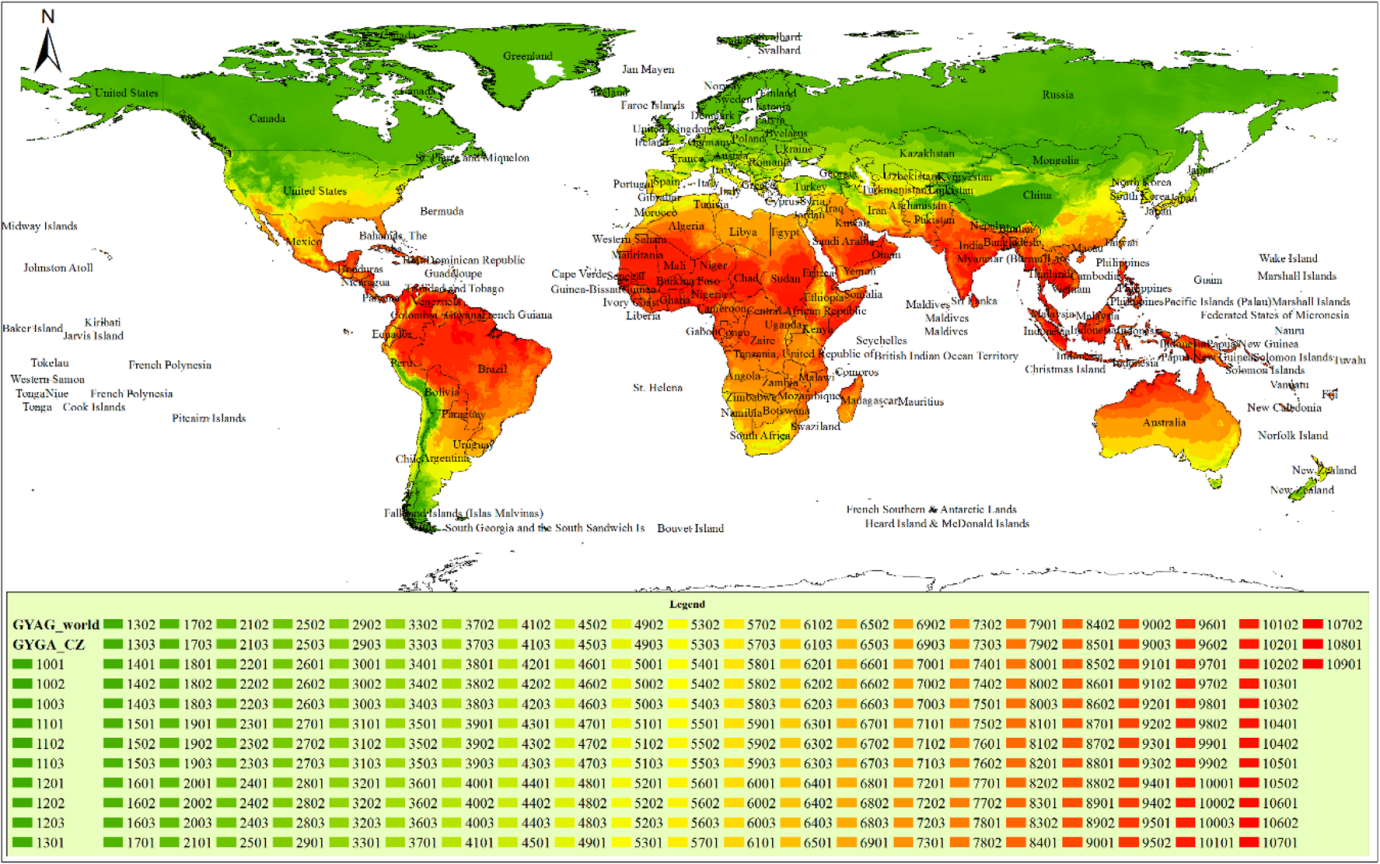
A world climate map based on the GYGA classification system, with green representing colder regions and red representing hotter regions. The climate code signifies a climate's specific traits, taking into account temperature (GDD), Temperature Seasonality, and Aridity Index. The map was generated utilizing ArcGIS software version 10.5.

The potential yield data is presented separately for various climate codes in different nations. The average potential yield was calculated based on the weighted area under cultivation in each climate code in every country and then generalized to comparable climate codes globally. In certain climates where data on potential yield was unavailable, the average potential yield was calculated for each temperature unit. This calculated value was then used as the potential yield. For instance, climate code 5903 had no information on its potential yield, so the average potential yield was determined based on climate codes 5003, 5103, 5203, and so on, with the same temperature unit of 5000. The result was then taken as the potential yield for climate code 5903. The Global Yield Gap Atlas map is displayed in Fig. 1.

### Soil zoning based on HC27

The soil data was acquired from IFPRI-Harvest Choice, as seen in Table 2^10^. The selection of soil types follows the GYGA protocol, which chooses the dominant soil based on its coverage of the wheat growing area within the RWS buffer zone. The soil that covers at least 50% of the wheat growing area within the buffer zone is considered the dominant soil. In the event that there is no dominant soil type within the RWS, the soils with a coverage of over 10% of the wheat growing area within the RWS are selected as the dominant soils.

**Table 2:** Global soil data from the IFPRI Harvest Choice study^10^.

Soil #	Soil code*	Depth (mm)	Albedo	Curve number	Drainage factor	Saturation limit (m3/m3)	Drained upper limit (m3/m3)	Lower limit (m3/m3)
1	HC1-Clay HF180	1800	0.05	85	0.2	0.458	0.405	0.233
2	HC2-Clay HF120	1200	0.05	85	0.2	0.458	0.405	0.233
3	HC3-Clay HF060	600	0.05	85	0.2	0.458	0.405	0.233
4	HC4-Clay MF180	1800	0.05	85	0.2	0.458	0.405	0.233
5	HC5-Clay MF120	1200	0.05	85	0.2	0.458	0.405	0.233
6	HC6-Clay MF060	600	0.05	85	0.2	0.458	0.405	0.233
7	HC7-Clay LF180	1800	0.05	85	0.2	0.458	0.405	0.233
8	HC8-Clay LF120	1200	0.05	85	0.2	0.458	0.405	0.233
9	HC9-Clay LF060	600	0.05	85	0.2	0.458	0.405	0.233
10	HC10-Loam HF180	1800	0.10	75	0.5	0.41	0.307	0.180
11	HC11-Loam HF120	1200	0.10	75	0.5	0.41	0.307	0.180
12	HC12-Loam HF060	600	0.10	75	0.5	0.41	0.307	0.180
13	HC13-Loam MF180	1800	0.10	75	0.5	0.41	0.307	0.180
14	HC14-Loam MF120	1200	0.10	75	0.5	0.41	0.307	0.180
15	HC15-Loam MF060	600	0.10	75	0.5	0.41	0.307	0.180
16	HC16-Loam LF180	1800	0.10	75	0.5	0.41	0.307	0.180
17	HC17-Loam LF120	1200	0.10	75	0.5	0.41	0.307	0.180
18	HC18-Loam LF060	600	0.10	75	0.5	0.41	0.307	0.180
19	HC19-Sand HF180	1800	0.15	65	0.75	0.365	0.169	0.073
20	HC20-Sand HF120	1200	0.15	65	0.75	0.365	0.169	0.073
21	HC21-Sand HF060	600	0.15	65	0.75	0.365	0.169	0.073
22	HC22-Sand MF180	1800	0.15	65	0.75	0.365	0.169	0.073
23	HC23-Sand MF120	1200	0.15	65	0.75	0.365	0.169	0.073
24	HC24-Sand MF060	600	0.15	65	0.75	0.365	0.169	0.073
25	HC25-Sand LF180	1800	0.15	65	0.75	0.365	0.169	0.073
26	HC26-Sand LF120	1200	0.15	65	0.75	0.365	0.169	0.073
27	HC27-Sand LF060	600	0.15	65	0.75	0.365	0.169	0.073

*Each soil code consists of three components: the soil type, soil depth, and fertility level represented by HF/MF/LF (high/medium/low fertility).

**Figure 2:**
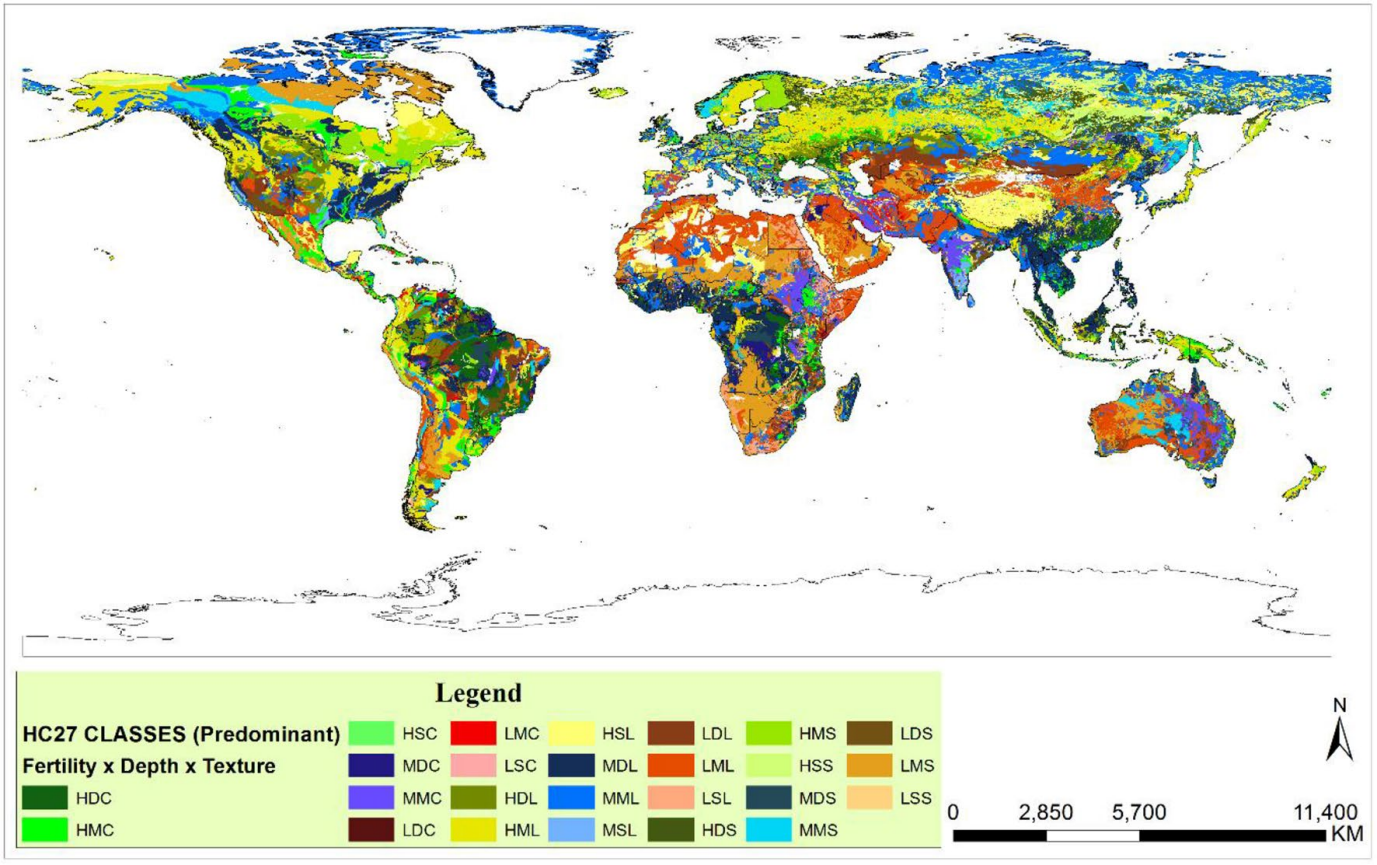
World soil map based on three parameters of fertility, soil depth and soil texture^10^. Fertility level represented by HF/MF/LF (high/medium/low fertility). Soil type is shown by S (Surface), M (Middle) and D (Deep). Soil texture displayed by C (Clay), L (Loam), and S (Sand). The soil data was obtained from IFPRI-Harvest Choice, and the map was generated utilizing ArcGIS software version 10.5.

According to a study by Nehbandani et al.^19^, the effectiveness of HC27 in the context of Iran was evaluated using the SSM-iCrop2 model. The results indicate that the soil database is suitable for Iran. The map was divided into different categories based on soil texture, soil depth, and fertility. It was separated into three groups of clay, silt, and sand based on soil texture, deep, medium, and shallow based on soil depth, and high, medium, and low fertility based on fertility (Table 2). Figure 2 shows the map of HC27 and the distribution of the soil types.

### Cropland area, actual yield, and production of irrigated and rainfed wheat

**Figure 3:**
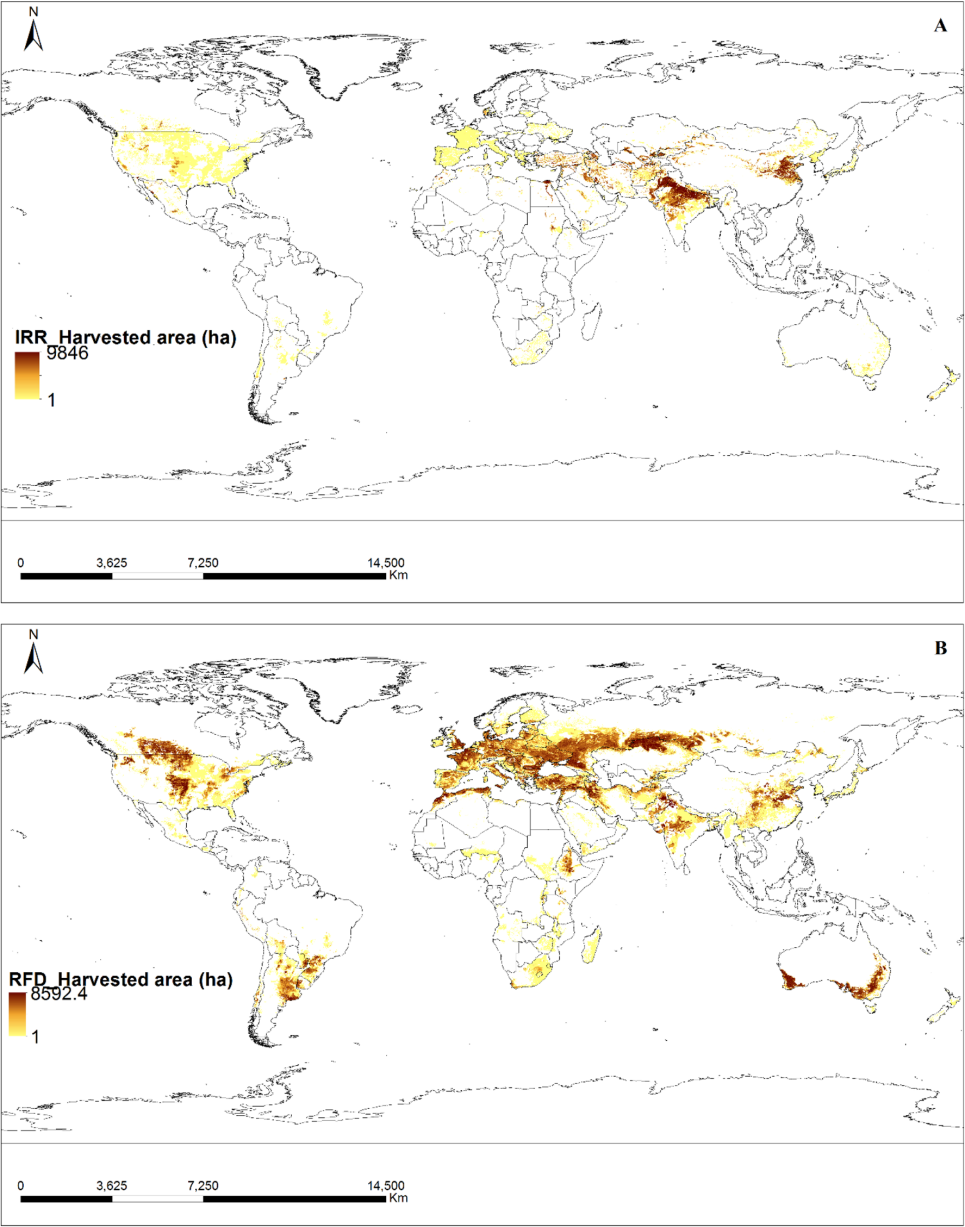
The cultivated area of irrigated (**A**) and rainfed (**B**) wheat: an overview. The solid brown color denotes the amount of cultivated area per pixel, with each pixel measuring 0.083333333 × 0.083333333 hectares. The source data were downloaded from The SPAM (Spatial Production Allocation Model) database and the map was generated utilizing ArcGIS software version 10.5.

**Figure 4:**
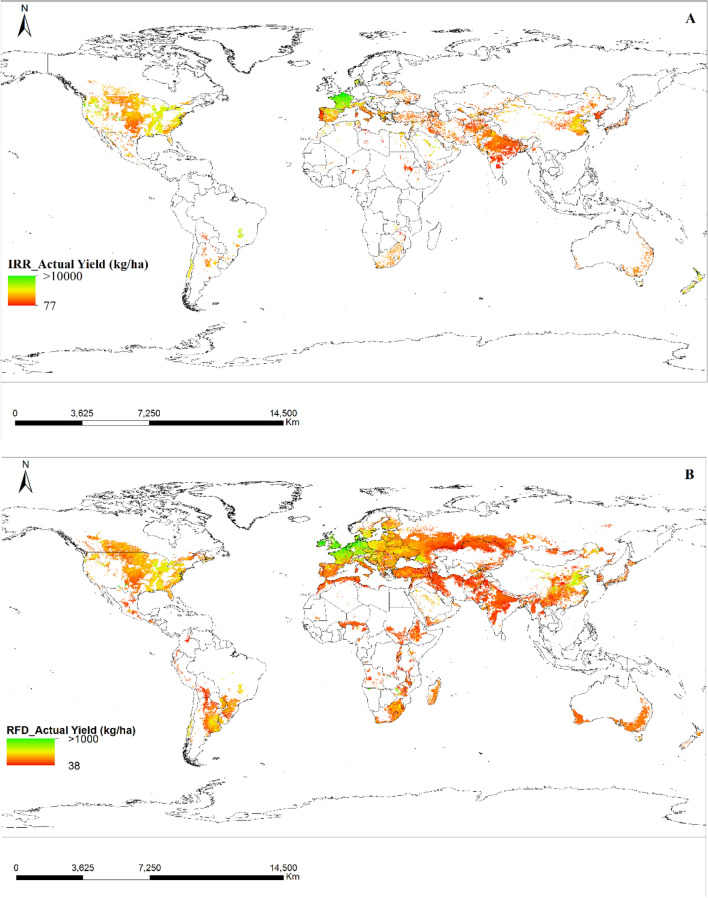
Illustrative map of the yield of irrigated (**A**) and rainfed (**B**) wheat. The green shade depicts the maximum output while the red shade indicates the minimum output. The source data were downloaded from The SPAM (Spatial Production Allocation Model) database and the map was generated utilizing ArcGIS software version 10.5.

**Figure 5:**
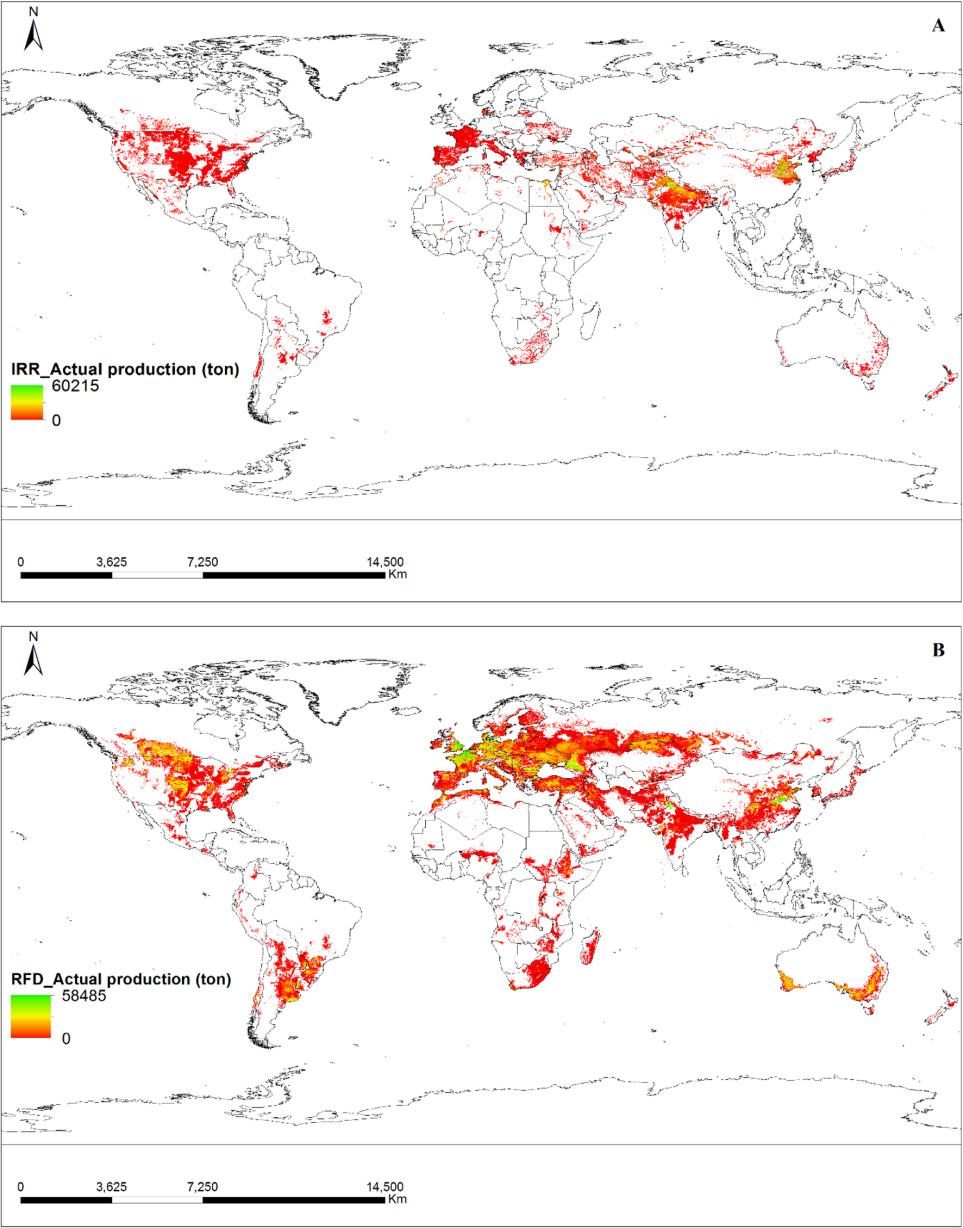
Illustrative map of the production of irrigated (**A**) and rainfed (**B**) wheat. The green shade depicts the maximum output while the red shade indicates the minimum output. The source data were downloaded from The SPAM (Spatial Production Allocation Model) database and the map was generated utilizing ArcGIS software version 10.5.

The maps of the global area of crops for irrigated and rainfed wheat were obtained from the SPAM (https://www.mapspam.info: SPAM 2010 v2.0 Global Data) website, as shown in Fig. 3. The crop area information was differentiated between the two conditions. Furthermore, additional maps and data, such as actual yield (Fig. 4) and production (Fig. 5) for both irrigated and rainfed conditions, were downloaded separately from the SPAM website. The maps were downloaded in shape file format from the SPAM website and were redrawn using ArcGIS software for this study.

The SPAM (Spatial Production Allocation Model) database serves as a widely trusted and valuable resource for comprehensive agricultural production data on a global scale. Its extensive coverage spans diverse geographical regions, encompassing various climatic and agro-ecological conditions, which proved instrumental for our research on irrigated and rainfed wheat lands worldwide. The database offers multi-dimensional data, and we specifically extracted information on cropland area, actual yield, and production of wheat under both irrigated and rainfed conditions. The spatial representation of data allowed us to create detailed and informative maps, aiding in the analysis of crop distributions and production patterns. While the SPAM database provides time series data, we primarily utilized the SPAM 2010 v2.0 Global Data, offering a snapshot of cropland area, actual yield, and production for the specific year of interest.

The process of creating thematic maps involved several sequential steps. Initially, data layers were gathered from various sources, including the GYGA Climate Map, SPAM database, and other relevant datasets, all in shape file format or compatible formats for GIS analysis. Next, relevant datasets were integrated, and attribute tables were joined to link specific data with geographic features, such as combining climatic information from the GYGA Climate Map with crop production data from the SPAM database. Quantitative data were then classified into thematic classes using appropriate methods like Natural Breaks (Jenks), Equal Interval, or Quantile to clearly represent patterns and variations on the thematic maps. After classification, suitable symbols, colors, or shading schemes were assigned to represent each thematic class, ensuring map readability and interpretability. Thematic maps were strategically organized to emphasize key findings and facilitate comprehension of spatial patterns and trends, accompanied by a legend explaining the symbology and thematic classes. Throughout the process, quality assurance checks were performed to ensure the accuracy and consistency of the thematic maps. This meticulous approach enabled the creation of thematic maps effectively illustrating the distribution and patterns of various agricultural parameters, such as climatic zones, cropland areas, actual yield, and production for irrigated and rainfed wheat lands.

## Results

### Agroclimatic zoning of irrigated wheat

The findings showed that the total global production of irrigated wheat is 239.6 million tons. This figure was calculated from a cropland area of 59.5 million hectares and an average yield of 4.02 t/ha. Among the climate zone codes, the highest production of irrigated wheat was observed in CZ #8002, 5203, 5303, 5403, 8102, 5503 and 7002 with an average production of 20.7, 14.3, 13.3, 11.11, 11.5, 99, and 9.8 million tons respectively. Other climate zones had a lower production rate (Table 3). The soil with the highest production was code #17, which is a loam soil with a depth of 120 cm and low fertility level. This particular soil type covers an area of 23.6 million hectares and is used for irrigated wheat cultivation. The soil type has an average yield of 4591 kg per hectare and produces nearly 106.8 metric tons of wheat (Table 4). Also, other soil types were including 14 (loam, deep of 120 cm, medium fertility), # 5(Clay, deep of 120 cm, medium fertility), # 26 (sand, deep of 120 cm, low fertility), # 13 (loam, deep of 180 cm, medium fertility) and # 11 (loam, deep of 120 cm, high fertility) which have the highest cropland area and production for irrigated wheat in the world scale (Table 4). According to Table 5, soil code #17 has the highest production and cropland area for irrigated wheat across all climate zones. The highest production with an average of 18.6 metric tons was recorded for climate zone # 8002 and soil code # 17 (Table 5).

**Table 3:** cropland area, yield, and production of irrigated wheat in each climate zone.

GYGA_CZ	Irrigated wheat area (MHA)	Irrigated wheat yield (t/ha)	Irrigated wheat production (Mt)
8002	5.3	4.12	20.7
5203	2.5	3.90	14.3
5303	2.2	4.32	13.3
5403	1.8	4.39	11.8
8102	2.4	3.70	11.5
5503	1.6	4.46	9.9
7002	1.8	4.39	9.8
9002	2.2	4.08	8.4
9302	2.6	2.96	7.1
8302	2.0	3.29	6.8
5003	1.4	3.84	5.7
Other	25.8	4.63	119.4
4027	59.5	4.03	239.6

**Table 4:** Cropland area, yield, and production of irrigated wheat in each soil type.

HC27	Irrigated wheat area (Mha)	Irrigated wheat yield (t/ha)	Irrigated wheat production (Mt)
17	23.6	4.59	106.8
14	12.8	4.13	41.9
5	6.4	4.58	23.5
26	3.1	4.62	16.2
13	4.3	4.91	15.8
11	3.1	4.31	12.3
Other	6.2	3.73	23.1
Sum	59.5	4.03	239.6

**Table 5:** Cropland area, yield, and production of irrigated wheat in each climate zone and soil type.

HC27	GYGA_CZ	Irrigated wheat area (Mha)	Irrigated wheat yield (t/ha)	Irrigated wheat production (Mt)
17	8002	4.8	4.39	18.6
17	5203	1.8	4.76	10.5
17	5303	1.6	6.36	9.5
17	7002	1.4	5.16	8.3
17	8102	1.5	4.47	7.6
17	9002	1.6	4.86	6.2
17	5403	0.8	5.56	5.2
17	5503	0.6	5.59	3.7
14	8502	1.4	2.29	3.4
26	5203	0.5	5.87	3.2
14	9302	1.1	3.03	3.2
17	6003	1.0	3.75	3.1
5	5503	0.5	4.60	3.0
13	8202	0.7	4.07	3.0
13	8302	0.8	3.57	3.0
17	4003	0.7	3.75	2.9
26	5303	0.4	6.06	2.7
14	9402	1.0	2.72	2.6
17	5003	0.6	3.42	2.6
14	8402	0.9	2.71	2.5
26	5403	0.4	6.12	2.5
14	9202	0.6	3.72	2.4
5	5403	0.3	4.96	2.4
17	4303	0.4	3.85	1.9
14	5503	0.3	4.52	1.7
13	8102	0.3	5.37	1.7
14	8602	0.6	2.39	1.6
27	9302	0.6	2.48	1.6
Other		32.1	3.71	119.1
Sum		59.5	4.03	239.6

### Agroclimatic zoning of rainfed wheat

The worldwide production of rainfed wheat is twice as much as irrigated wheat, with a total of 410 million metric tons produced from 160 million hectares of cropland. The average yield of rainfed wheat is 2.55 t/ha. According to agro-ecological zoning results, the best production is found in six specific climate zones, identified as #3702, #3802, #1303, #1203, #1403, and #3602, which produce 17, 12.1, 11.9, 11.3, 11.3 and 10.2 million metric tons respectively (Table 6). Additionally, the greatest yield was recorded in climate code 3802, whereas the lowest yield was found in the climate code with a higher growing day degree. Based on soil type analysis, the highest yield was seen in soil codes #11, #10, #14, #17, #13, #5, #2, #1, and #26 with an average of 81.4, 61.0, 60.9, 37.1, 35.4, 27.7, 26.4, 23.8 and 15.7 Mt (Table 7). Among various soil types, soil code #10 had the highest yield. In terms of the combination of climate zone code and soil type, the highest yield with an average of 5.9 Mt was observed in climate #3702 and soil code #13 (as stated in Table 8).

**Table 6:** Cropland area, yield, and production of rainfed wheat in each climate zone code.

GYGA_CZ	Rainfed wheat area (Mha)	Rainfed wheat yield (t/ha)	Rainfed wheat production (Mt)
3702	2.4	3.51	17.0
3802	1.7	3.89	12.1
1303	6.4	1.45	11.9
1203	7.3	1.38	11.7
1403	5.3	1.58	11.3
3602	1.6	3.50	10.2
4602	1.8	3.07	9.5
2802	1.5	3.21	9.5
3502	2.0	3.30	9.1
2702	1.8	3.30	9.0
4402	2.6	2.29	8.3
1503	3.6	1.53	7.7
2603	2.9	2.22	7.6
3403	3.0	1.82	7.4
4702	1.5	2.73	7.3
5503	1.7	2.48	7.3
2203	5.1	1.37	7.3
4502	1.7	2.78	7.3
4403	1.9	2.33	7.1
4303	2.4	2.13	6.9
5403	2.1	2.51	6.8
6102	3.9	1.57	6.6
3503	2.1	2.08	6.4
Other	66.2	3.10	205.4
Sum	160.2	2.56	410.0

**Table 7:** Cropland area, yield, and production of rainfed in each soil type.

HC27	Rainfed wheat area (Mha)	Rainfed wheat yield (t/ha)	Rainfed wheat production (Mt)
11	32.9	2.10	81.4
10	26.8	2.61	61.0
14	22.4	2.07	60.9
17	16.8	2.13	37.1
13	7.5	2.52	35.4
5	10.9	2.47	27.7
2	10.1	2.00	26.4
1	8.9	1.92	23.8
26	6.4	2.55	15.7
Other	17.4	2.33	40.5
Sum	160.2	2.56	410.0

**Table 8:** Cropland area, yield, and production of rainfed in each soil type.

HC27	GYGA_CZ	Rainfed wheat area (Mha)	Rainfed wheat yield (t/ha)	Rainfed wheat production (Mt)
13	3702	0.7	3.81	5.9
11	1303	3.0	1.43	5.3
11	1203	2.8	1.48	5.2
11	1403	2.4	1.69	5.1
10	2203	3.3	1.67	5.0
10	1303	2.2	1.91	4.8
10	3403	1.7	2.58	4.4
10	1203	3.2	1.68	4.3
11	2603	1.5	2.54	4.0
1	4403	0.9	2.28	3.8
13	3802	0.4	4.54	3.6
10	3503	1.2	2.89	3.6
14	3702	0.5	3.27	3.3
14	2802	0.5	3.39	3.3
14	4702	0.6	2.36	3.3
11	1503	1.5	1.89	3.2
10	2103	1.6	1.94	3.2
14	4602	0.6	3.07	3.1
10	1403	1.2	1.96	3.0
14	2702	0.6	2.44	3.0
1	3303	1.2	2.13	2.7
17	5503	0.5	3.58	2.6
10	2303	1.1	2.03	2.6
1	4303	0.7	2.52	2.5
17	6002	1.8	1.56	2.5
13	3602	0.3	4.12	2.4
14	4102	0.9	2.00	2.4
14	3802	0.4	3.59	2.4
17	5403	0.5	2.63	2.4
2	3702	0.3	3.98	2.4
10	4203	0.8	3.09	2.4
5	5503	0.5	2.96	2.3
11	3503	0.6	2.77	2.2
13	2802	0.3	4.55	2.2
10	4103	0.7	2.83	2.0
10	2403	1.0	1.84	2.0
2	1503	0.9	2.79	2.0
11	4402	0.5	2.45	1.9
Other		116.8	2.46	287.7
Sum		160.2	2.56	410.0

### Evaluation of climatic parameters in irrigated condition

**Figure 6:**
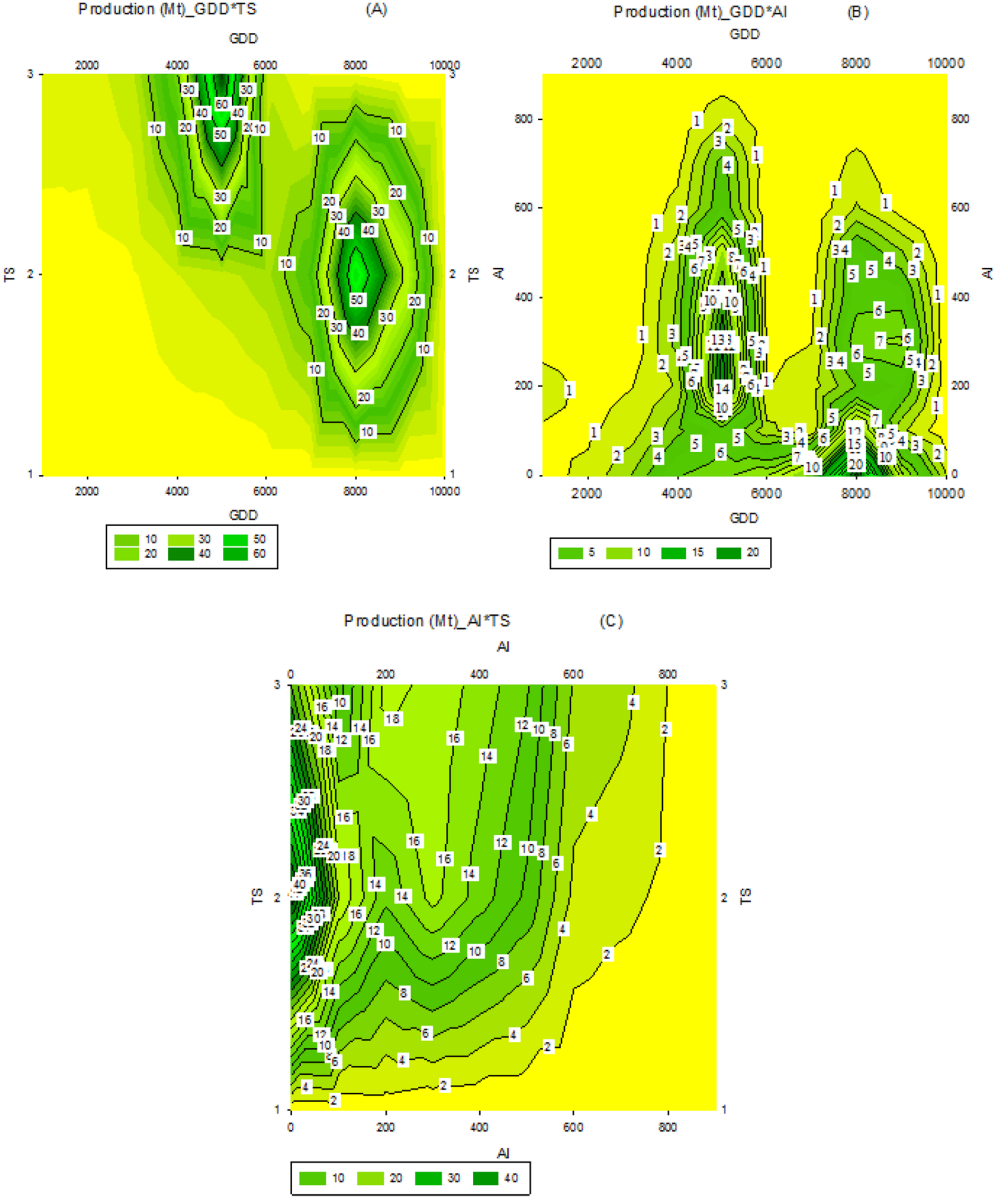
Relationship between growing degree day (GDD) and temperature seasonality (TS) (**A**), GDD and aridity index (AI) (**B**), and AI × TS (**C**) on the irrigated wheat production in different climatic regions of the world.

The results of the study on the connection between growth degree days (GDD) and temperature seasonality (TS) revealed that the highest yields under irrigation were achieved in two scenarios—either high GDD (8000) with medium TS (2) or medium GDD (5000) with high TS (3). The production under these conditions was estimated to be between 50 to 60 Mt (Fig. 6A). The research on the relationship between Aridity Index (AI) and GDD showed that the production was concentrated in areas with GDD of 5000 and AI of 300, resulting in a production of around 10 to 14 Mt (Fig. 6B). Additionally, the joint impact of AI and TS was evaluated and it was found that high yields were generated in areas with TS of 2 and AI between 100 to 200, and similar results were seen in areas with TS of 3 and AI of 400 (Fig. 6C).

### Evaluation of climatic parameters in rainfed condition

**Figure 7:**
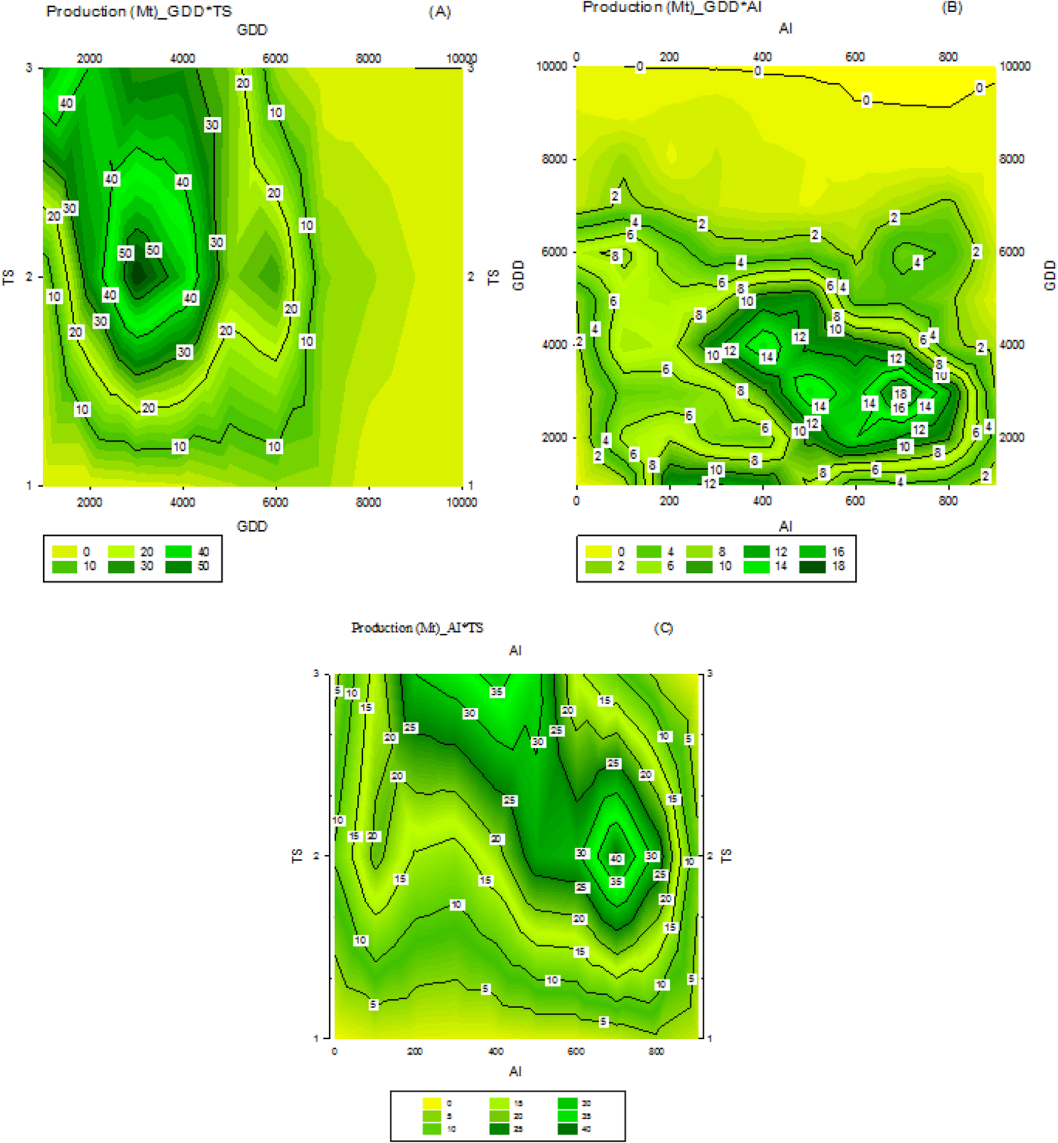
The relationship between the growth degree day (GDD) and the aridity index (AI) (**A**), the aridity index and temperature seasonality (**B**), and GDD × TS (**C**) in rainfed wheat production.

According to the analysis of the climate codes shown in Fig. 7, regions with low GDD (Fig. 7A) and high AI are ideal for rainfed wheat cultivation because these regions receive more rainfall than average and have cooler air temperatures than average. The concentration of production is seen in the range of 600 to 800 in terms of AI and code #2 in terms of TS. A code of 1 for TS indicates minimal differences between night and day temperatures, while codes 2 and 3 indicate a greater difference (Fig. 7B). The best conditions for wheat production are identified as areas with code 3000 for GDD and TS #2 (Fig. 7C), which align with the ideal growing conditions for rainfed wheat.

## Discussion

The quality and availability of land and water resources, together with important socio‐economic and institutional factors, are essential for food security^20^. The ability to cultivate crops, referred to as crop cultivation potential, is determined by the maximum production that can be achieved under specific agro-climatic, soil, and terrain conditions, with a given level of agricultural inputs and management practices^21^. The Agro-Ecological Zones (AEZ) method is built upon the principles of land evaluation as outlined by Fischer et al.^1^. The AEZ idea was first introduced by the United Nations Food and Agriculture Organization (FAO). Since then, the FAO and the International Institute for Applied Systems Analysis (IIASA) have worked together to further refine and apply the AEZ approach. This has involved updating the supporting databases and software tools. The latest version of the Global AEZ (GAEZ v3.0), features significant improvements over its 2002 predecessor^22^ and includes updated data and a wider application of the methodology.

This research utilized climate maps, soil information, and the extent of irrigated and rainfed wheat fields to identify the main cultivation areas of wheat. The climate code reported on the Global Yield Gap Atlas (GYGA) website and the soil type were taken into consideration. The agroecological zoning method, a commonly used approach for identifying the primary and potential expansion areas for crops and other products, was applied^23^. Additionally, by analyzing the factors that define climate codes, such as the degree-day-growth (GDD), temperature seasonality (TS), and aridity index (AI) and their impact on wheat production, it was found that the correlation between irrigated and rainfed agriculture and these parameters is strong, and that these parameters play a significant role in determining which regions are suitable for expansion.

The aridity index is calculated by dividing the amount of precipitation by the annual evapotranspiration^24^. A higher aridity index signifies a region with a more humid climate. It has been established that in rainfed conditions, access to water for the plant, or humidity, is more crucial for yield production than Growing Degree Days (Fig. 7A). Even when temperature seasonality is increased, meaning the length of the plant's growth period is extended due to autumn cultivation, it does not surpass the importance of moisture. This demonstrates that the success of rainfed land cultivation relies mainly on rainfall, with factors like TS and GDD having secondary influence on yield. Research has shown that when it comes to dryland wheat cultivation in areas with low moisture, it is better to extend the growth period through autumn cultivation, rather than cultivate the crop in warm and temperate regions, as this is preferred over increasing GDD^25^.

In irrigated agriculture, the dryness of the climate is less important compared to Growing Degree Days (GDD) as shown in Fig. 6A. Irrigation reduces the impact of Aridness Index (AI) on wheat production. The amount of GDD may vary in different regions, with lower levels in cold areas and higher levels in warm or hot climates, but it does not impact yield in irrigated conditions. This has been confirmed by other research studies^26^. It is worth mentioning that while cold climates may result in slightly higher production due to a longer growth period, irrigation offers the ability to manage the microclimate of the farm, enhancing production management. The significance of TS in irrigation conditions becomes apparent when compared to AI. This indicates that the priority in irrigation farming is focused on TS, while in rainfed farming, AI holds a special importance.

A further inquiry that arises is whether the largest area of wheat cultivation through irrigation and rainfed methods takes place in the soil types or climate zones that occupy the largest space globally. In terms of soil type, the answer is almost always yes in both irrigation and rainfed circumstances (Table 9). However, when examining climate zones, there is less alignment between the dominant global climate zones and the areas designated for production, particularly in rainfed conditions. These findings suggest that soil limitations have a greater impact on the expansion of cultivation than climate conditions^27^. As previously stated, irrigation can significantly control the environmental conditions for wheat cultivation. Hence, one of the ways to enhance global wheat production is by advancing its irrigation-based farming^28,29^. Wheat is an ancient plant with a vast and complex genome, making it possible to improve its cultivation due to its adaptability to environmental factors^30,31^.

**Table 9:** Area of each soil code and GYGA-CZs.

Soil code	%	Soil code	%	GYGA-CZs	%	GYGA-CZs	%
11	14.36	5	3.23	7002	5.103	1603	2.021
14	13.24	23	3.14	1803	4.795	10,002	2.002
17	11.33	19	2.12	1503	3.525	9002	1.910
26	10.49	20	2.05	9901	3.513	1303	1.624
13	9.66	21	1.61	1903	3.510	8801	1.584
12	6.75	18	1.53	1703	2.978	9801	1.471
16	4.10	25	1.48	8002	2.593	8701	1.455
2	3.95	4	1.29	1403	2.289	4003	1.348
1	3.85	Sum	97.50	10,001	2.208	Sum	46.01
10	3.33	Other	2.5	6002	2.087	Other	53.98

Considering the production limitations in each region, we address the question of whether soil or climate parameters serve as the limiting factors. To do so, we conduct separate evaluations for each continent under both rainfed and irrigated conditions. In rainfed wheat production, Asia (5%) and North America (5.5%) faces the most significant limitation when considering GDD (Growing Degree Days). On the other hand, Europe (34%), Africa (26.5%), and South America (23%) are regions that boast the highest percentage of area with suitable GDD for rainfed wheat cultivation. Under irrigation conditions, the continents of North America (28%) and Australia (29%) show the lowest limit for the percentage of cultivated area receiving suitable Growing Degree Days (GDD). On the other hand, Asia and South America experience more limitations under these conditions.

In rainfed conditions, the aridity index serves as a limiting factor across all continents with varying intensities. However, temperature seasonality is generally not a restricting factor for wheat production under this circumstance. South America (74.4%), Africa (67.5%) and Asia (57%) stand out as the most desirable continents concerning the percentage of cultivated area with suitable Aridity Index (AI) for irrigation-based cultivation. Taking both factors into account, North America, in general, will have a higher percentage of land suitable for wheat production. In other words, these two factors will result in fewer restrictions for wheat cultivation in this continent under irrigation conditions.

In rainfed conditions, soil resource limitations are apparent in Australia, with Africa and Asia experiencing a lesser degree of restriction. On the other hand, under irrigation conditions in Australia, Europe, and South America, they encounter relatively less suitable soil resources. In this article, we will not discuss the yield and production gap as it is beyond the scope of our focus. Based on the results, it is evident that Asia and Australia should prioritize soil reclamation programs, while Africa, Europe, and South America should concentrate on adopting suitable genotypes to overcome uncontrollable conditions. This approach is not exhaustive and acknowledges the need for detailed studies in each country. Nonetheless, it offers a general perspective on the priorities to enhance wheat production.

## Conclusion

The findings of the study indicated that the growth of irrigated wheat demands a higher amount of growth degree days (GDDs) compared to wheat grown without irrigation. Hence, the cultivation of irrigated wheat mainly occurs in regions with higher temperature units (GDDs). Additionally, the cultivars of irrigated wheat need more temperature units to complete their growth cycle compared to rainfed wheat cultivars. With regards to the aridity index, irrigated wheat is more suitable for drier climates compared to wheat grown through rainfall. In areas where the rainfall is insufficient to meet the water requirements for wheat growth, most of the production of irrigated wheat takes place.

In irrigated environments, as these crops are mostly cultivated in arid regions, the highest yields are observed in areas with a significant temperature differential between summer and winter. Conversely, in humid environments, the highest production is observed in regions with a lower temperature differential and a higher degree of precipitation. Given that irrigated wheat is primarily grown in dry areas, the development of drought-resistant cultivars with early maturity is crucial to reduce the need for irrigation. On the other hand, in regions with high rainfall, resistance to pests and diseases is an important factor to consider in the cultivation of this crop. There are various other factors that impact the growth of this crop, and while they were beyond the scope of this study, diseases and pests cannot be disregarded. Additionally, the preference for a particular crop variety varies among regions with similar climate and soil conditions. However, this study demonstrates the potential for expanding the cultivation of this main crop by identifying the key climate and soil characteristics of the main wheat production areas.

Finally, the priority for the wheat yield improvement program in each region depends on whether the limitation in production is due to uncontrollable environmental factors or more manageable soil conditions. Understanding this distinction in smaller areas like a country or region can be beneficial for prioritizing wheat production improvement programs.

### Future scope of research

As the present study sheds light on the agro-ecological zones of irrigated and rainfed wheat lands around the world, several avenues for future research emerge to deepen our understanding of global agricultural dynamics. The following points outline potential areas for further investigation:

- Climate Change Impact: Given the ongoing changes in global climate patterns, future research should focus on assessing the impact of climate change on the identified agro-ecological zones. Investigating variations in Growing Degree Days (GDD), Temperature Seasonality, and Annual Aridity Index (AI) under different climate scenarios will provide valuable insights into the vulnerability of specific regions to climate-induced shifts in agricultural productivity.
- Comparative Crop Productivity: Expanding the scope of this research to encompass additional crops and comparing their productivity between irrigated and rainfed lands within the identified agro-ecological zones will offer comprehensive insights into crop suitability and potential yield gaps. Such comparative analyses will aid policymakers and farmers in optimizing resource allocation and fostering sustainable agricultural practices.
- Integration of Remote Sensing Data: Future research can explore the integration of remote sensing data and advanced machine learning algorithms to enhance the accuracy of crop mapping and yield estimation within the identified agro-ecological zones. Leveraging satellite imagery and cutting-edge technology will provide a more refined understanding of spatial crop distribution and support precision agriculture practices.
- Socio-economic Factors: Understanding the socio-economic factors influencing agricultural practices and yield variability within each agro-ecological zone is critical for developing targeted strategies for sustainable agricultural development. Researchers can delve into factors such as access to resources, technology adoption, and policy frameworks to tailor interventions specific to regional needs.
- Interdisciplinary Approaches: Encouraging interdisciplinary collaborations between agronomists, climate scientists, economists, and policymakers will yield comprehensive insights into the complex interactions between climate, soil, and agricultural productivity. Integrating diverse expertise will foster holistic approaches to address global food security challenges.

### Key points for researchers

In addition to the future research possibilities, the present study yields important points for researchers to consider in the context of global agricultural patterns:

- The comprehensive analysis of agro-ecological zones provides a foundation for understanding the geographic distribution of suitable wheat cultivation areas, facilitating regional-specific agricultural planning.
- The thematic maps generated in this study offer valuable visual representations of agro-climatic zones, which can aid policymakers and agricultural planners in decision-making processes.
- The integration of the GYGA Climate Map and SPAM database establishes a robust framework for assessing crop distribution and productivity, which can be extended to study other crops and regions.
- The study highlights the significance of considering both climatic and soil factors in defining agro-ecological zones, emphasizing the need for tailored approaches to address diverse agricultural challenges.
- Future research focusing on the relationships between climatic variables and crop productivity will lead to targeted interventions and innovations in agriculture, contributing to sustainable global food systems.

By considering these future research directions and key points, researchers can build upon the findings of this study and collectively work towards addressing pressing challenges in global agriculture.

## Data Availability

The datasets generated and/or analysed during the current study are available from the corresponding author (m.chaichi@areeo.ac.ir) on reasonable request.
